# *In cellulo* phosphorylation of DNA double-strand break repair protein XRCC4 on Ser260 by DNA-PK

**DOI:** 10.1093/jrr/rry072

**Published:** 2018-09-22

**Authors:** Ali Reza Amiri Moghani, Mukesh Kumar Sharma, Yoshihisa Matsumoto

**Affiliations:** 1 Laboratory for Advanced Nuclear Energy, Institute of Innovative Research, Tokyo Institute of Technology, 2-12-1-N1–30, Ookayama, Meguro-ku, Tokyo, Japan; 2 Department of Zoology, SPC Government College, Ajmer, Rajasthan, India

**Keywords:** DNA double-strand break repair, non-homologous end joining, XRCC4, DNA-dependent protein kinase, phosphorylation-specific antibody, radiosensitivity

## Abstract

XRCC4 is one of the core factors for DNA double-strand break (DSB) repair through non-homologous end joining (NHEJ). XRCC4 is phosphorylated by DNA-dependent protein kinase (DNA-PK), with Ser260 and Ser320 (Ser318 in the alternatively spliced form) being the major phosphorylation sites *in vitro*. It was recently reported that Ser320 is phosphorylated by DNA-PK in response to DNA damage; however, it is currently unclear whether Ser260 is phosphorylated *in cellulo* in response to DNA damage. Herein, we generated an antibody against XRCC4 phosphorylated on Ser260 and examined its phosphorylation status via Western blotting. XRCC4 Ser260 phosphorylation increased after irradiation with 30–300 Gy of γ-rays and was suppressed by DNA-PK inhibitor but not by ATM inhibitor. Moreover, XRCC4 Ser260 phosphorylation decreased in DNA-PKcs–deficient cells. These observations indicate that XRCC4 Ser260 is phosphorylated by DNA-PK *in cellulo*. The XRCC4^S260A^ mutant reversed the high radiosensitivity of XRCC4-deficient M10 cells to a similar level to that of wild-type XRCC4. However, the clonogenic survival of cells expressing the XRCC4^S260A^ mutant was slightly but significantly lower than that of those expressing wild-type XRCC4. In addition, XRCC4^S260A^-expressing cells displayed a significantly greater number of γ-H2AX foci than XRCC4^WT^-expressing cells 4 h after 1 Gy irradiation and without irradiation. The present results suggest a potential role of XRCC4 Ser260 phosphorylation by DNA-PK in DSB repair.

## INTRODUCTION

DNA double-strand breaks (DSBs) are considered the most severe type of DNA damage. DSBs are generated exogenously by ionizing radiation or chemicals, including a subset of anti-cancer agents, and endogenously by replication errors or oxidative stress. DSBs also result from recombination, including meiotic recombination in reproductive organs and V(D)J recombination in immune cells. There are two major mechanisms underlying DSB repair in eukaryotic cells: non-homologous end joining (NHEJ) and homologous recombination [1]. Core NHEJ machinery comprises Ku70, Ku86 (also known as Ku80) [2, 3], DNA-dependent protein kinase catalytic subunit (DNA-PKcs) [4–7], XRCC4 [8], DNA ligase IV (LIG4) [9, 10], XRCC4-like factor (XLF; also known as Cernunnos) [11, 12], and Paralog of XRCC4 and XLF (PAXX; also known as XLS for XRCC4-like small molecule) [13–15]. Ku70 and Ku86 form a heterodimer, bind first to the DNA end and recruit DNA-PKcs. The ternary complex of Ku70, Ku86 and DNA-PKcs is termed DNA-PK and is believed to act as a sensor for DSBs. XRCC4 is compactly associated with LIG4, which finally ligates two DNA ends, and is required for its stabilization and nuclear localization [9, 10, 16–18]. XRCC4, XLF and PAXX are structurally related to one another and to a centrosome-regulating protein SAS-6 [13, 14]. As NHEJ is also involved in V(D)J recombination to generate diverse immunoglobulins and T cell receptors in the immune system, animals deficient in NHEJ factors exhibit immunodeficiency. XRCC4 mutations were recently identified in patients exhibiting microcephaly and dwarfism; however, unexpectedly, these patients presented normal immune function [19–24].

XRCC4 is phosphorylated by DNA-PK *in vitro* [9, 25, 26] and also in cultured cells after exposure to ionizing radiation or treatment with DNA-damaging agents [27, 28]. Mass spectrometry analyses of XRCC4 phosphorylated by DNA-PK *in vitro* revealed that Ser260 and Ser320 (initially termed as Ser318 according to an alternatively spliced form) of XRCC4 are the major phosphorylation sites [29–31]. However, it remains unclear whether these sites are phosphorylated by DNA-PK *in cellulo*. Moreover, XRCC4 mutants lacking these phosphorylation sites appeared to be functionally normal in terms of radiosensitivity, V(D)J recombination, and NHEJ reactions in a cell-free system [29, 30]. Thus, XRCC4 phosphorylation by DNA-PK at these sites was considered unnecessary for DSB repair. It has been recently reported that XRCC4 Ser320 is phosphorylated in response to DNA damage by DNA-PK [32]. This study sought to determine (i) whether Ser260 of XRCC4 is phosphorylated *in cellulo* and (ii) whether XRCC4 phosphorylation contributes to cell survival after irradiation.

## MATERIALS AND METHODS

### Generation of an XRCC4 Ser260 phosphorylation-specific antibody

An XRCC4 Ser260 phosphorylation-specific antibody, α-XRCC4-pS260, was generated in accordance with previous studies that generated phosphorylation-specific antibodies against p53 Ser15, p53 Ser37, p53 Ser46 [33] and XRCC4 Ser320 (α-XRCC4-pS320) [32].

Peptides XRCC4-S260-C (DDSIISSLDVTDIC, corresponding to XRCC4 254–266 with a cysteine appended at C-terminus) and XRCC4-S260-P (the same sequence as XRCC4-S260-C but the serine corresponding to Ser260 has been phosphorylated) were synthesized by Greiner BIO ONE. Rabbits were immunized and antisera were collected by Protein Purify (Isezaki, Gunma, Japan).

In the aforementioned studies [32, 33], antisera were first passed through a column with unphosphorylated peptide several times and then a column with phosphorylated peptide. In this study, the order of the columns was exchanged because we noticed that antibodies reacting with unphosphorylated protein could be more efficiently eliminated by passing through the unphosphorylated peptide column after elution from the phosphorylated peptide column.

### Enzyme-linked immunosorbent assay

The specificity and titer of the antibody was examined via enzyme-linked immunosorbent assay (ELISA). Fifty microliters of peptide solution (1 μg/ml in 200 mM NaHCO_3_-NaOH, pH 9.2) was applied into each well of an ELISA plate (3801–096, AGC Techno Glass, Tokyo, Japan). After standing for 1 h at room temperature, the peptide solution was eliminated and the wells were washed thrice with PBS(–) containing 0.05% v/v Tween 20 (T-PBS). Wells were then filled with 200 μl of PBS(–) containing 0.5% w/v bovine serum albumin (BSA-PBS) for blocking. After allowing the reaction plates to stand for 1 h at room temperature, BSA-TBS was eliminated and the wells were washed thrice with T-PBS. Thereafter, 25 μl of serially diluted antibody solution was applied into each well. After allowing the plates to stand for 1 h at room temperature, the antibody solution was eliminated and the wells were washed four times with T-PBS and once with Tris-buffered saline [TBS; 20 mM Tris-HCl (pH 7.6), 0.9% w/v NaCl]. Thereafter, 50 μl of horseradish peroxidase (HRP)-conjugated swine anti-rabbit immunoglobulins (P0399, Dako) were added in each well. After allowing the plates to stand for 1 h at room temperature, the antibody solution was eliminated and the wells were washed four times with T-PBS and once with TBS. Thereafter, 100 μl of substrate solution (ELISA POD substrate A.B.T.S. kit, Nacalai Tesque) was added to each well. The absorbance was measured using the iMark plate reader (Bio-Rad), at 405 nm.

### *In vitro* phosphorylation

Human full length XRCC4 protein with a hexa-histidine (6xHis) tag at the C-terminus was expressed in *E. coli*. Full-length XRCC4 cDNA was obtained via polymerase chain reaction (PCR) from the cDNA pool of human T-cell leukemia MOLT-4 cells, using a pair of primers X4FL-F and X4FL-R (Table 1), and cloned into pET41d vector (Novagen) between restriction sites of *Nco*I and *Xho*I (Takara). Point mutations were introduced via PCR, as described [34], using the primers listed in Table 1. The entire XRCC4 open reading frame was sequenced by Fasmac (Atsugi, Kanagawa, Japan) and found to be accurate. The resultant plasmids were transformed into *E. coli* HIT-21 competent cells (RBC Biosciences, New Taipei, Taiwan). When the optical density spectrophotometrically at 600 nm (O.D._600_) approached 0.6, isopropyl β-D-1-thiogalactopyranoside was added to the culture medium at a final concentration of 1 mM. The bacterial cells were harvested 4–5 h later via centrifugation at 7000*g* for 5 min and resuspended in extraction buffer [20 mM sodium phosphate buffer (pH7.4), 500 mM NaCl, and 25 mM imidazole]. The suspension was sonicated and centrifuged at 20 000*g* for 20 min. The supernatant was passed through a 0.22-μm filter and then injected into a His-Trap column (1 ml, GE Healthcare). After loading the entire lysate, 10 ml of extraction buffer was injected to eliminate any unbound material. Thereafter, bound material was eluted with 4 ml of the extraction buffer with imidazole at increasing concentrations, i.e. 50 mM, 200 mM, 350 mM and 500 mM, and XRCC4 protein was most abundant in the eluate with 200 mM imidazole. The buffer was exchanged with XRCC4 storage buffer [50 mM sodium phosphate buffer (pH7.4), 20 mM glycerol and 2 mM dithiothreitol] by using a desalting chromatography column PD-10 (GE Healthcare).

**Table 1.** Polymerase chain reaction primers used in this study

Name	Sequence
X4-FL-F	TTAAGCC*ATG*GAGAGAAAAA TAAGCAGAAT CC
X4-FL-R	AGACTCGAGA ATCTCATCAA AGAGGTCTTC TGGG
S259A-F	ATTATTGCAA GTCTTGATGT CACTGAT
S259A-R	AAGACTTGCA ATAATGGAAT CATCTTT
S260A-F	ATTTCAGCAC TTGATGTCAC TGATATT
S260A-R	ATCAAGTGCT GAAATAATGG AATCATC
S260D-F	ATTTCAGACC TTGATGTCAC TGATATT
S260D-R	ATCAAGGTCT GAAATAATGG AATCATC
S259/260A-F	ATTATTGCAG CACTTGATGT CACTGAT
S259/260A-R	AAGTGCTGCA ATAATGGAAT CATCTTT

In the sequences of X4-FL-F and X4-FL-R, the *Nco*I site and the *Xho*I site, respectively are underlined, and the initiation codon in X4-FL-F is italicized. Double-underlined characters represent those corresponding to the mutated codons.

DNA-PK was purified from human leukemia MOLT-4 cells via successive column chromatography [35]. XRCC4 and DNA-PK were incubated in the reaction buffer containing 20 mM HEPES-NaOH (pH 7.2), 50 mM KCl, 5 mM MgCl_2_, 50 μM ATP, 1 mM dithiothreitol and phosphatase inhibitor cocktail (Sigma-Aldrich) at 37°C for 30 min and then analyzed via Western blotting.

### Cell culture

Human cervical carcinoma cell line HeLa was obtained from the Japanese Collection of Research Bioresources (JCRB) Cell Bank. Human glioma cell lines M059K and M059J, the latter of which lacks DNA-PKcs [7], were obtained from American Type Culture Collections. These cells were cultured as described previously [32]. Human lymphoblastoid TK6-derived wild-type cell line (TSCE5) and DNA-PKcs knock-out cell line were obtained from Dr Shunichi Takeda and Dr Hiroyuki Sasanuma [36]. Murine leukemia L5178Y-derived, XRCC4-deficient cell line M10, harboring a nonsense mutation in *XRCC4* (c.A370T, p.R124X) [37, 38], was obtained from RIKEN Cell Bank with the permission of Dr Koki Sato. M10 and its transformants (see below) were used as described previously [39].

Human XRCC4 cDNA, integrated into p3XFLAG-CMV-10 vector (Sigma-Aldrich), was constructed as described previously [40]. Point mutations were introduced via PCR as described previously [34], using the primers listed in Table 1. The entire XRCC4 open reading frame was sequenced by Fasmac. Wild-type or mutated XRCC4 cDNA was transfected into M10 cells, using a Neon Transfection system (Invitrogen). Two days after transfection, cells were plated in 0.2% agarose-containing RPMI1640 medium supplemented with 15% v/v bovine calf serum, 100 units/ml penicillin, 100 μg/ml streptomycin, 10 μM β-mercaptoethanol and 0.8 mg/ml G418, as p3XFLAG-CMV-10 harbors the neomycin resistance gene as a selection marker. Two weeks after plating, visible colonies were selected and expanded to obtain stably transformed clones.

### Cell irradiation and treatment with inhibitors

Cells were irradiated using a ^60^Co γ-ray source in Tokyo Institute of Technology (222 TBq in February 2010). The dose rate was measured using an ionizing chamber–type exposure dosemeter C-110 (Oyo Giken, Tokyo, Japan). DNA-PK inhibitor, NU7441 [41] (Tocris Bioscience), and ATM inhibitor, KU55933 [42] (EMD Biochemicals), were dissolved in dimethyl sulfoxide at 5 mM and added to the culture medium at a final concentration of 10 μM at 2 h before irradiation.

### Western blotting

Western blotting was performed as described previously [32]. Anti-XRCC4 rabbit polyclonal antibody, α-XRCC4, and XRCC4 Ser320 phosphorylation-specific antibody, α-XRCC4-pS320, were generated previously [32]. Anti-PCNA rabbit polyclonal antibody (FL261) was purchased from Santa Cruz Biotechnology. Anti-rabbit immunoglobulins swine polyclonal antibody conjugated with horseradish peroxidase (P0399) was purchased from DAKO. The immunocomplexes were developed using Western Lightning ECL-Pro reagent (PerkinElmer) or WesternSure Chemiluminescent Western Blot reagent (LI-COR) and the images were captured using C-Digit Blot Scanner (LI-COR). Protein Ladder One Triple-color Broad Range (Nacalai Tesque) was used as the standard molecular mass marker.

### Immunoprecipitation

One hour after γ-ray irradiation, cells were harvested via centrifugation at 290*g* for 5 min at 4°C and washed twice in PBS(–). The cell pellet was suspended in IP Lysis Buffer [50 mM Tris-HCl (pH 7.6), 150 mM NaCl and 0.5% Triton X-100] supplemented with 1% v/v, respectively, of protease inhibitor cocktail and phosphatase inhibitor cocktail (Nacalai Tesque) at a density of 1–2 × 10^7^ cells/ml, and after culturing at 4°C for 30 min, the cells were centrifuged at 20 000*g* for 7 min at 4°C.

The supernatant was incubated with 10 μg of α-XRCC4 or control rabbit immunoglobulin (Medical & Biological Laboratories) for 2 h at 4°C and then with a 50 μl suspension of Pure Proteome Protein G Magnetic Beads (Merck Millipore) for 10 min at room temperature. The immunocomplexes were captured magnetically then washed thrice with IP Lysis Buffer. The beads were suspended in 30–40 μl of 2XSDS-PAGE Loading Buffer [125 mM Tris-HCl (pH 6.8), 4% w/v sodium lauryl sulfate, 20% v/v glycerol, 5% v/v β-mercaptoethanol, 0.02% w/v Bromophenol Blue and 0.01% Crystal Violet], heated at 100°C for 10 min and then subjected to Western blotting. Importantly, secondary antibody was not used because it might react intensely with α-XRCC4, which was used for immunoprecipitation. Primary antibodies α-XRCC4 and α-XRCC4 Ser320 were conjugated with peroxidase, using Ab-10 Rapid Peroxidase Labeling Kit (Dojindo Molecular Technologies).

### Measurement of cellular radiosensitivity

Cellular radiosensitivity was measured in terms of colony-forming ability in soft agarose, as described previously [38]. Five hundred microliters of appropriately diluted cell suspension was mixed with 12 ml of 0.2% w/v agarose-containing RPMI1640 medium, supplemented with 15% v/v bovine calf serum, 100 units/ml penicillin, 100 μg/ml streptomycin and 10 μM β-mercaptoethanol, and dispensed into three plastic dishes of 60 mm in diameter. After culturing for 10–12 d, visible colonies were counted. Plating efficiency was calculated as the number of colonies divided by the number of plated cells. Surviving fraction was calculated as the plating efficiency of irradiated cells divided by the plating efficiency of unirradiated cells. Statistical significance was determined via the one-sided Welch’s *t*-test.

### Immunofluorescent staining

Immunofluorescent staining of γ-H2AX was performed in accordance with our previous study [18] with several modifications. M10-CMV, M10-XRCC4^WT^ and M10-XRCC4^S260A^ were attached on a glass slide via centrifugation at 100*g* for 5 min. Cells were fixed with 4% paraformaldehyde-containing PBS(–) and permeabilized with 0.5% Triton X-100–containing PBS(–). Slides were incubated first with 5% bovine serum albumin–containing PBS(–) (BSA-PBS) for 1 h at room temperature for blocking and then with anti-γ-H2AX rabbit polyclonal antibody (Merck Millipore, 05–636) in BSA-PBS at 4°C overnight. After washing four times with 0.05% Tween 20-containing PBS (T-PBS), slides were incubated with Alexa Fluor 594-conjugated anti-mouse IgG(H+L) goat polyclonal antibody (Invitrogen, A-11 032) for 2 h at room temperature. Hereafter, slides were maintained in the dark. After washing five times with T-PBS, slides were stained with 100 ng/ml of 4′,6-diamidino-2-phenylindole dihydrochloride (DAPI) in Fluorescent Mounting Medium (DAKO) and observed using an inverted fluorescent microscope IX-71 (Olympus). Fluorescent images were analyzed using ImageJ software and γ-H2AX foci were automatically detected and enumerated. Statistical significance was determined using one-sided Welch’s *t*-test.

## RESULTS

### Generation of α-XRCC4-pS260

**Fig. 1.**
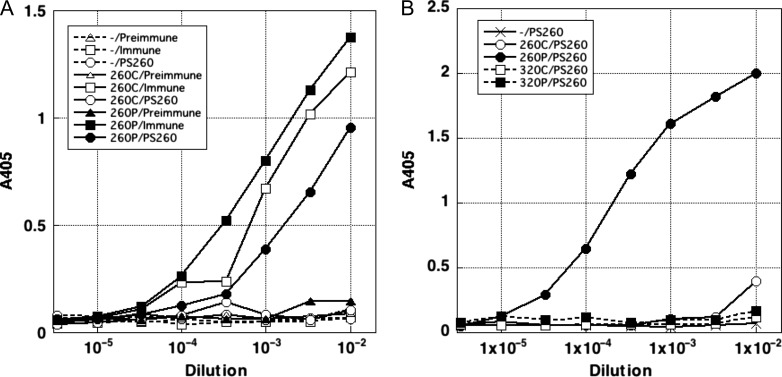
The specificity of α-XRCC4-pS260 examined via an enzyme-linked immunosorbent assay (ELISA). (A) ELISA of preimmune and immune rabbit sera and purified antibody α-XRCC4-pS260. (B) ELISA of α-XRCC4-pS260 for XRCC4 Ser260 and Ser320 phosphorylation. –: wells coated with buffer without peptide; 260C, 260P, 320C and 320P: wells coated with XRCC4-S260-C, XRCC4-S260-P, XRCC4-S320-C and XRCC4-S320-P peptides, respectively; PS260: α-XRCC4-pS260.

The reactivity of the preimmune or immune rabbit sera and purified antibody α-XRCC4-pS260 to unphosphorylated and phosphorylated XRCC4 Ser260 was examined via ELISA. Preimmune serum did not exhibit detectable reactivity to XRCC4-S260-P or XRCC4-S260-C peptides (Fig. 1A). The immune serum reacted with XRCC4-S260-P and XRCC4-S260-C, although the reactivity to phosphorylated peptide was somewhat stronger. Purified α-XRCC4-pS260 reacted with XRCC4-S260-P but not with XRCC4-S260-C. In another ELISA, we also used phosphorylated and unphosphorylated peptides, XRCC4-S320-P and XRCC4-S320-C, respectively, corresponding to XRCC4 Ser320 (Fig. 1B). Purified α-XRCC4-pS260 did not show any detectable reactivity to XRCC4-S320-P and XRCC4-S320-C. These results indicate that α-XRCC4-pS260 enable the detection of XRCC4 Ser260 phosphorylation, distinguishing it from Ser320 phosphorylation.

### Phosphorylation of XRCC4 Ser260 by DNA-PK in vitro

**Fig. 2.**
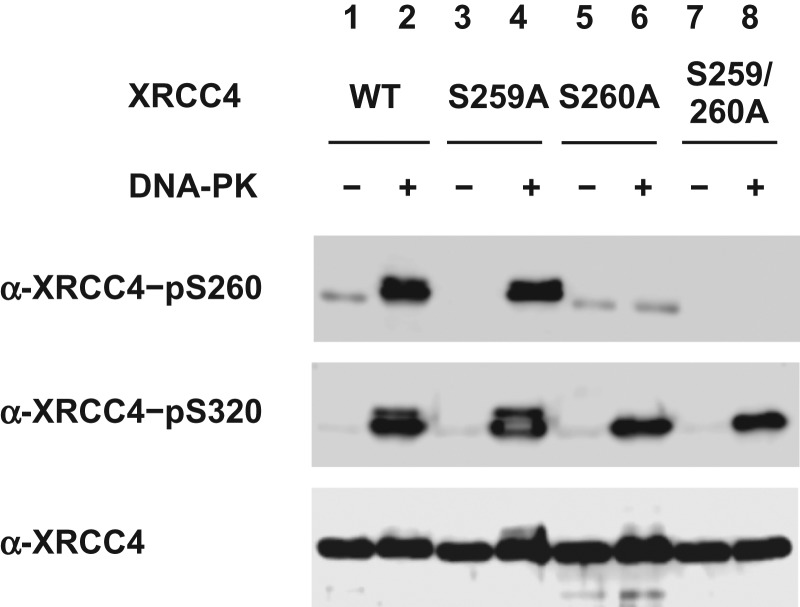
Phosphorylation of XRCC4 Ser260 and Ser320 by DNA-dependent protein kinase (DNA-PK) *in vitro*. Wild-type (WT) or mutated XRCC4 proteins were phosphorylated by DNA-PK *in vitro* and the reaction products were analyzed via Western blotting using α-XRCC4-pS260 and α-XRCC4-pS320 antibodies.

XRCC4, which was expressed in and purified from *E. coli*, was phosphorylated *in vitro* by DNA-PK, which was purified from cultured human cells. α-XRCC4-pS260 and α-XRCC4-pS320 reacted with wild-type XRCC4 after treatment with DNA-PK but not after mock treatment without DNA-PK (Fig. 2, lanes 1 and 2). The reaction of α-XRCC4-pS260 was diminished in XRCC4^S260A^ (lanes 5 and 6) and in XRCC4^S259/260A^ (lanes 7 and 8), but was not affected in XRCC4^S259A^ (lanes 3 and 4). However, α-XRCC4-pS320 did react with XRCC4^S260A^ and XRCC4^S259/260A^, although the intensity was somewhat decreased and the upper band, probably representing a multiply phosphorylated form, was no longer observed. These results indicate that Ser260 is phosphorylated by DNA-PK *in vitro*, concurrent with previous studies using mass spectrometry [29–31], and also substantiated the specificity of α-XRCC4-pS260 toward Ser260-phosphorylated XRCC4.

### Phosphorylation of XRCC4 Ser260 in cellulo

**Fig. 3.**
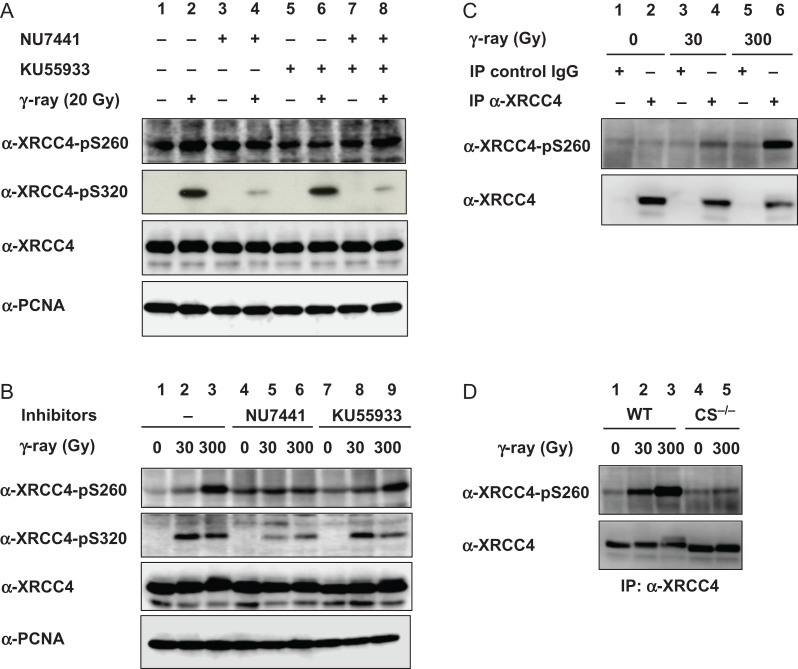
XRCC4 Ser260 and Ser320 phosphorylation in cultured human cells after γ-ray irradiation. (A and B) HeLa cells harvested 30 min (A) or 1 h (B) after irradiation with the indicated dose of γ-rays in the presence or absence of 10 μM of specific inhibitors for DNA-dependent protein kinase (DNA-PK) and ATM (NU7441 and KU55933, respectively). (C and D) HeLa cells (C) and wild-type or DNA-PKcs^–/–^ TK6 cells (D) were harvested 1 h after irradiation with the indicated dose of γ-rays and subjected to immunoprecipitation with α-XRCC4 or control rabbit IgG, as indicated. The immunoprecipitant was examined by western blotting using α-XRCC4-pS260 or α-XRCC4 antibodies that had been conjugated with peroxidase.

We initially examined the phosphorylation status of XRCC4 Ser260 and Ser320 in HeLa cells after 20 Gy γ-ray irradiation in the presence and absence of NU7441 and KU55933, which are specific inhibitors for DNA-PK and ATM, respectively (Fig. 3A). As reported previously, Ser320 phosphorylation was mostly undetectable in unirradiated cells (lane 1); however, it was greatly enhanced after irradiation (lane 2). NU7441, but not KU55933, greatly diminished the Ser320 phosphorylation (compare lanes 2, 4 and 6). On the other hand, Ser260 phosphorylation was mostly unchanged with or without 20 Gy γ-ray irradiation and in the presence or absence of inhibitors (lanes 1 to 8). Thus, XRCC4 Ser260 might be constitutively phosphorylated, whereas Ser320 is phosphorylated by DNA-PK in response to DNA damage.

However, the phosphorylation of XRCC4 Ser260 increased after irradiation with a higher dose, i.e. 300 Gy, of γ-rays (Fig. 3B, lane 3). This was reversed by NU7441, but not by KU55933 (compare lanes 6 and 9 with lane 3). The autophosphorylation of DNA-PKcs on Ser2056 was suppressed by NU7441 but not by KU55933 and showed similar dose dependency to the phosphorylation of XRCC4 Ser260 (Figure S1). However, Chk2 Ser19 phosphorylation was suppressed by KU55933, but not by NU7441. These results indicate that at this extremely high dose, XRCC4 Ser260 is phosphorylated by DNA-PK. To confirm the identity of the band, we performed immunoprecipitation followed by Western blotting (Fig. 3C). The band of XRCC4 Ser260 phosphorylation was detected in anti-XRCC4 immunoprecipitant of 300 Gy γ-irradiated cells and, to a lesser extent, in that of 30 Gy γ-irradiated cells.

To clarify the role of DNA-PKcs, we next examined the phosphorylation status of XRCC4 Ser260 in DNA-PKcs^–/–^ cells derived from human lymphoblastoid TK6 via Western blotting after immunoprecipitation with anti-XRCC4 antibody. The absence of DNA-PKcs in DNA-PKcs^–/–^ cells was confirmed via Western blotting (Figure S2). XRCC4 Ser260 phosphorylation increased after 30 Gy or 300 Gy γ-irradiation in wild-type TK6; however, it was mostly diminished in DNA-PKcs^–/–^ cells (Fig. 3D). These results indicate that XRCC4 Ser260 was phosphorylated by DNA-PK in response to γ-irradiation.

### Function of XRCC4 Ser260 phosphorylation–defective mutant in DSB repair

**Fig. 4.**
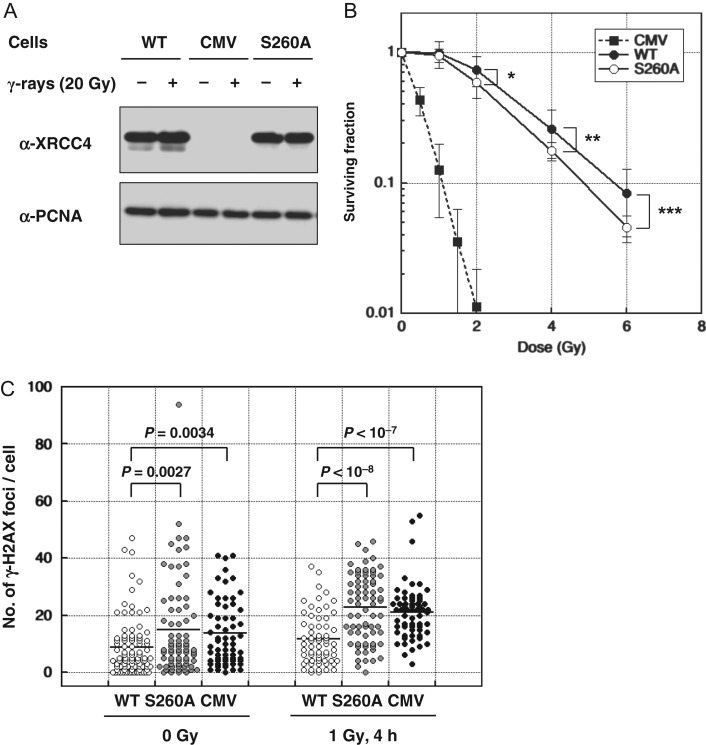
(A) Western blotting analysis of wild-type and S260A-mutated XRCC4 in M10-derived transformants. Proliferating cell nuclear antigen (PCNA) is shown as the loading control. (B) Radiosensitivity of M10-XRCC4^WT^, M10-XRCC4^S320A^ and M10-CMV cells measured by colony formation assay in 0.2% w/v agarose. Each symbol represents the average surviving fraction obtained from 8–10 repeated experiments, with the error bar indicating standard deviation. The *P* value was calculated by applying the data to Welch’s one-sided *t*-test. *P = 0.049, **P = 0.023, ***P = 0.017. (C): Number of γ-H2AX foci per cell in M10-XRCC4^WT^, M10-XRCC4^S320A^ and M10-CMV cells. Cells were harvested 4 h after 1 Gy irradiation or without irradiation and subjected to immunostaining using anti-γ-H2AX antibody and fluorescent-labeled secondary antibody. More than 60 cells were examined for each group. Each circle represents the number of foci in a single cell, and the horizontal bar shows the average number of foci per cell in the group. The *P*-value was calculated by applying the data to Welch’s one-sided *t*-test.

To investigate the functional significance of XRCC4 Ser260 phosphorylation, XRCC4^S260A^, in which Ser260 was converted to alanine to disable phosphorylation, was introduced into the M10 cell line, which lacks endogenous XRCC4, and the stable transformant, M10-XRCC4^S260A^ was established. The radiosensitivity of M10-XRCC4^S260A^ was measured via a colony-formation assay and compared with that of wild-type XRCC4 and vector control transformants, M10-XRCC4^WT^ and M10-CMV, respectively [39]. Notably, the expression level of XRCC4^S260A^ was comparable with that of XRCC4^WT^ (Fig. 4A). Similar to M10-XRCC4^WT^, M10-XRCC4^S260A^ exhibited much higher survival after irradiation than M10-CMV (Fig. 4A). Thus, XRCC4^S260A^ mutants could reverse the high radiosensitivity of XRCC4-deficient M10 cells to a similar extent to wild-type XRCC4, indicating that these mutants were mostly functional. Nevertheless, the surviving fraction of M10-XRCC4^S260A^ was consistently lower than that of M10-XRCC4^WT^ (Fig. 4B). The difference in the surviving fraction between M10-XRCC4^S260A^ and M10-XRCC4^WT^ was small but significant (*P* < 0.05), as determined via a one-sided Welch’s *t*-test at 2, 4 and 6 Gy (Fig. 4B).

To evaluate the DSB repair function of XRCC4^S260A^, we examined γ-H2AX foci via immunofluorescence staining. M10-XRCC4^S260A^ and M10-CMV showed significantly greater γ-H2AX foci than M10-XRCC4^WT^ 4 h after 1 Gy irradiation and without irradiation (Fig. 4C).

These results indicate that XRCC4^S260A^ might have incomplete function in DSB repair.

## DISCUSSION

XRCC4 was phosphorylated by DNA-PK *in vitro* and in cultured cells, and Ser260 and Ser320 were identified as the major phosphorylation sites *in vitro*. As it was reported recently that XRCC4 Ser320 is phosphorylated by DNA-PK in response to DNA damage in cultured cells [32], this study aimed to investigate XRCC4 Ser260 phosphorylation status *in cellulo* and its role in DSB repair.

First, we generated a phosphorylation-specific antibody for XRCC4 Ser260 and examined its phosphorylation status *in cellulo* with or without irradiation. XRCC4 Ser260 underwent constitutive and radiation-inducible phosphorylation. Radiation-inducible phosphorylation was inhibited by DNA-PK inhibitor and was diminished in DNA-PKcs^–/–^ cells, indicating that it would be mediated by DNA-PK. Constitutive phosphorylation was not affected by DNA-PK inhibitor. To date, however, no kinase, other than DNA-PK, is known to phosphorylate XRCC4 Ser260. Thus, the identity of the kinase responsible for constitutive phosphorylation of XRCC4 Ser260 is currently unknown. Furthermore, phosphorylation of Ser260 and Ser320 might be differentially regulated, although both of these serine residues are phosphorylated by DNA-PK in response to radiation. Ser320 phosphorylation increased with radiation dose between 1 and 20 Gy [32]; however, it remained unchanged or somewhat decreased after 300 Gy irradiation as compared with 30 Gy irradiation in this study. On the other hand, Ser260 phosphorylation increased after 300 Gy irradiation as compared with 30 Gy irradiation; the underlying reason is unclear, requiring further investigation.

Further, we constructed a XRCC4 mutant, XRCC4^S260A^, and examined its functionality in terms of its ability to complement hyper-radiosensitivity of XRCC4-deficient cells. Cells expressing this mutant were slightly but significantly more radiosensitive than cells expressing wild-type XRCC4. Furthermore, XRCC4^S260A^-expressing cells showed a greater number of γ-H2AX foci with or without irradiation. These results indicate that XRCC4^S260A^ does not function completely in DNA repair, suggesting a potential role for XRCC4 Ser260 phosphorylation by DNA-PK in DSB repair. With an increase in the number of γ-H2AX foci in M10-XRCC4^S260A^ compared with in M10-XRCC4WT, even without irradiation, XRCC4 Ser260 phosphorylation might also be involved in the repair of naturally occurring DSBs, probably generated via replication errors or oxidative stresses. The precise role of XRCC4 Ser260 phosphorylation in DSB repair remains unclear. It was recently shown that changing Ser260 together with other seven potential DNA-PK/ATM phosphorylation sites in XRCC4 into aspartate facilitated its dissociation from DNA [43]. When this octuple aspartate mutant was co-expressed with the hexatruple aspartate mutant of XLF in XRCC4- and XLF-disrupted cells, the cellular sensitivity toward DNA damaging agents was even greater than that of cells expressing alanine-substituted mutants [43].

In conclusion, the present study shows that XRCC4 Ser260 is phosphorylated by DNA-PK *in cellulo* in response to radiation. The present results also show that the XRCC4 mutant lacking this phosphorylation site is mostly but not completely functional in DSB repair, in terms of cell survival after irradiation. The present results indicate a possibility that the phosphorylation of XRCC4 Ser260 by DNA-PK plays a potential role in DSB repair, which warrants further clarification in future studies.

## Supplementary Material

Supplementary Data

Supplementary Data

Supplementary Data
