# Supplementary figures and images for: In cellulo phosphorylation of DNA double-strand break repair protein XRCC4 on Ser260 by DNA-PK
Source: J Radiat Res. 2018 Sep 22;59(6):700–8. doi: 10.1093/jrr/rry072 (PMC6251426; doi:10.1093/jrr/rry072)

## Slide 1
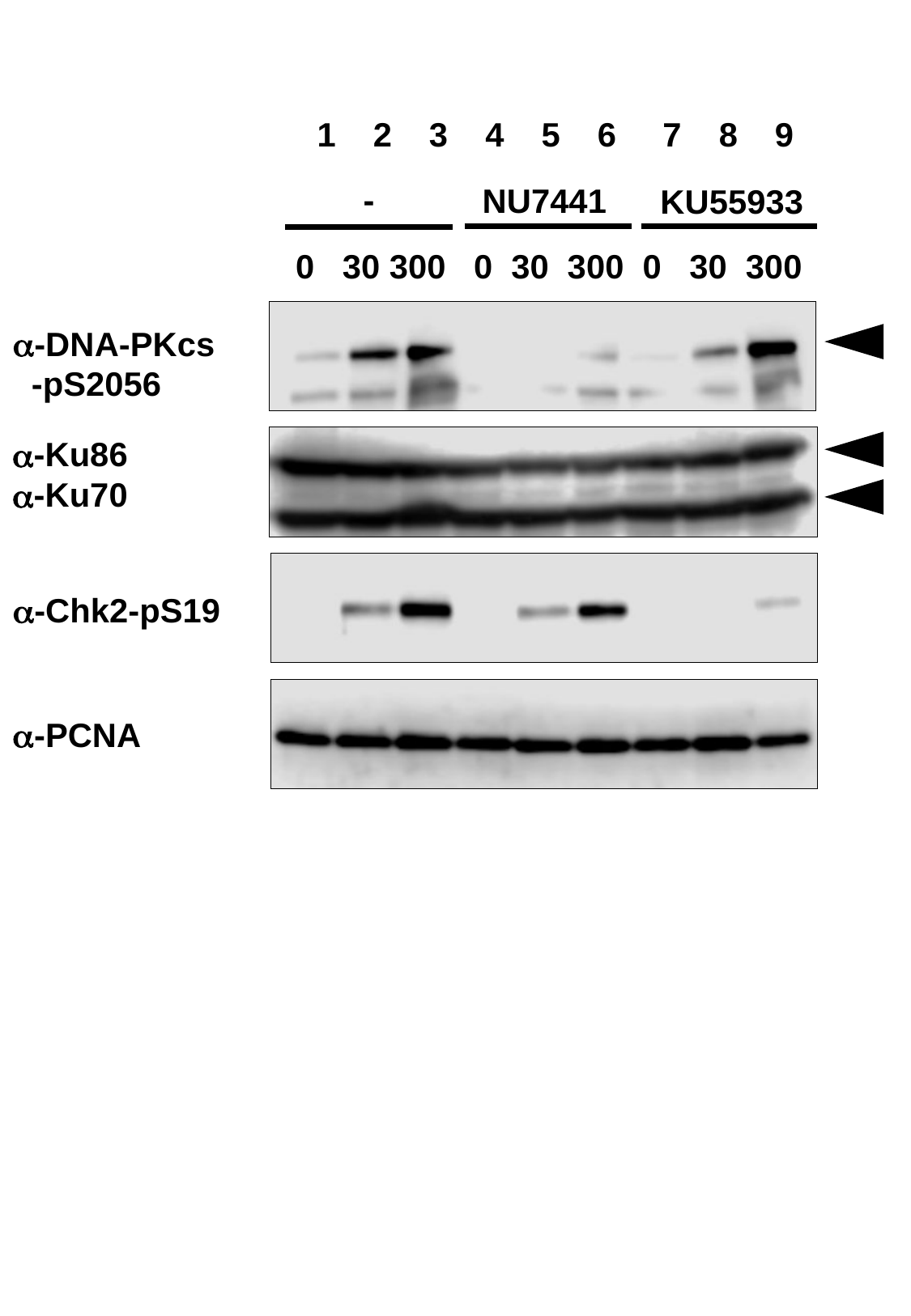

1 2 3 4 5 6 7 8 9
-
NU7441
KU55933
 0 30 300 0 30 300 0 30 300
a-DNA-PKcs
 -pS2056
a-Ku86
a-Ku70
a-Chk2-pS19
a-PCNA

Supplement: Supplementary Data [file rry072_figures1.pptx]

## Slide 1
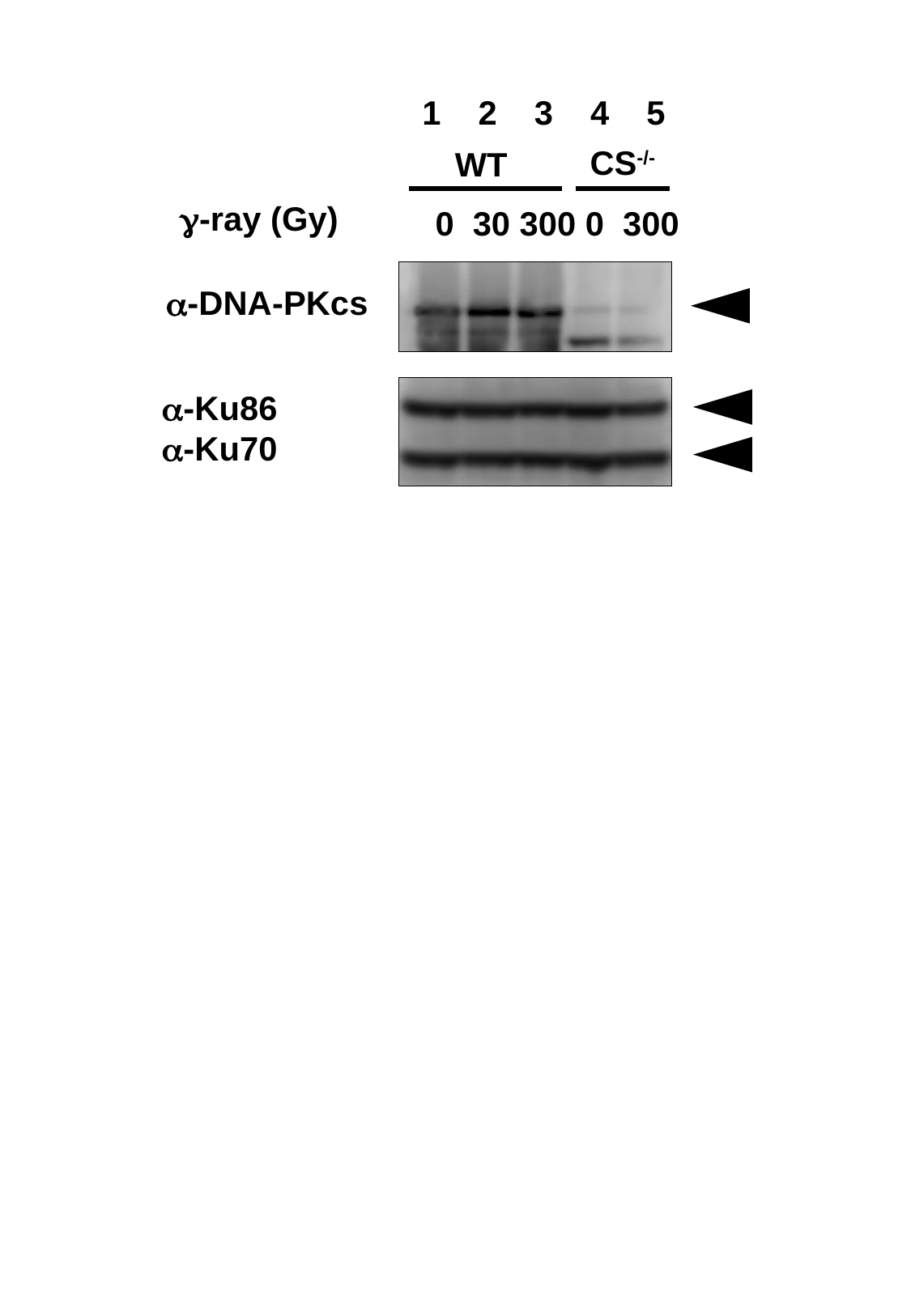

1 2 3 4 5
CS-/-
WT
g-ray (Gy)
0 30 300 0 300
a-DNA-PKcs
a-Ku86
a-Ku70

Supplement: Supplementary Data [file rry072_figures2_r1.pptx]
