# Supplementary material for: In cellulo phosphorylation of DNA double-strand break repair protein XRCC4 on Ser260 by DNA-PK
Source: J Radiat Res. 2018 Sep 22;59(6):700–8. doi: 10.1093/jrr/rry072 (PMC6251426; doi:10.1093/jrr/rry072)
Supplement: Supplementary Data [file rry072_legends_to_supplementary_figure.docx]

**Legends to Supplementary Figure**

Figure S1. Effects of inhibitors for DNA-dependent protein kinase (DNA-PK) and ATM on DNA-PKcs autophosphorylation and Chk2 phosphorylation. As shown in Figure 3B, HeLa cells harvested 1 h after irradiation with the indicated dose of γ-ray in the presence or absence of 10 μM of specific inhibitors for DNA-PK and ATM (NU7441 and KU55933, respectively). Ku86, Ku70 and PCNA are also shown as loading control.

Figure S2. Expression of DNA-PKcs in TK6-derived wild-type (TSCE5) and DNA-PKcs^-/-^ cells. Ku86 and Ku70 are also shown as loading control.
